# Optimizing auditory stimulation timing in NREM sleep using brain–heart rhythms: continuous phase analysis and multidimensional phase-locking

**DOI:** 10.1093/sleep/zsag017

**Published:** 2026-01-28

**Authors:** Sepehr Sardooeinasab, Massimiliano de Zambotti, Fiona C Baker, Mohamad Forouzanfar

**Affiliations:** Department of Electrical Engineering, École de Technologie Supérieure, 1100 Notre-Dame St W, Montreal, QC H3C 1K3, Canada; Center for Health Sciences, SRI International, 333 Ravenswood Ave, Menlo Park, CA 94025, United States; Center for Health Sciences, SRI International, 333 Ravenswood Ave, Menlo Park, CA 94025, United States; Center for Health Sciences, SRI International, 333 Ravenswood Ave, Menlo Park, CA 94025, United States; Centre de Recherche de l’Institut Universitaire de Gériatrie de Montréal (CRIUGM), Queen Mary Road, Montreal, QC H3W 1W5, Canada; Department of Systems Engineering, École de Technologie Supérieure, 1100 Notre-Dame St W, QC H3C 1K3, Canada

**Keywords:** brain–heart coupling, acoustic stimulation, slow oscillations, slow-wave activity, electroencephalogram, heart rate

## Abstract

**Study Objectives:**

Auditory stimulation during non-rapid eye movement sleep effectively enhances slow oscillations and slow-wave activity (SWA) when precisely timed to certain phases of the slow oscillation. However, timing precision remains a core challenge. Recent evidence suggests that heart rate components may provide effective complementary timing cues. This study examined which heart rate phases are associated with stronger stimulation responses using continuous phase analysis and evaluated a multidimensional phase-comparison approach that integrates heart rate and EEG slow oscillation phases.

**Methods:**

Polysomnography recordings from 133 adolescents were analyzed. Auditory tones were delivered randomly every 15–30 s during non-rapid eye movement (NREM) sleep. Instantaneous phases of EEG slow oscillation (~0.8 Hz) and heart rate components in the low-frequency (0.04–0.15 Hz) and high-frequency (0.15–0.4 Hz) bands were extracted for continuous phase analysis. Tone-evoked slow oscillation amplitude and slow-wave activity were further compared across three phase-locking strategies: unimodal (slow oscillation-only or heart rate-component-only) and combined (EEG–heart rate).

**Results:**

Responses were largest when tones occurred near the heart rate-low-frequency up-peak and heart rate-heart rate down-peak. Phase analyses showed that tones occurring at the optimal heart rate component phases were accompanied by increases in slow oscillation amplitude by up to ~22 μV and SWA by 12 per cent, indicating that peripheral signals can serve as strong, independent timing cues. Slow oscillation-only phase-locking also produced notable effects (~18 μV slow oscillation amplitude, 19% SWA increase). Combining slow oscillation and heart rate phases yielded the greatest effects, with increases of ~38 μV in slow oscillation amplitude and 32 per cent in SWA.

**Conclusions:**

Oscillatory phases derived from heart rhythms provide effective timing information that may be useful for closed-loop auditory stimulation and reflect brain–heart coupling during sleep. A multidimensional phase-based approach that integrates EEG slow oscillations with instantaneous heart rate phases may support more precise control and stronger enhancement of deep sleep than unimodal approaches, suggesting a new framework for closed- loop neuromodulation.

## Teaser

**Statement of Significance** The current research identified oscillatory phases that were associated with stronger responses to applying auditory stimulation to enhance deep sleep, based on main heart rate oscillation components. It indicated that heart rate component phases may provide useful timing information for sleep interventions both independently and in combination with brain slow oscillations. The findings suggest that synchronization of tones based on both brain and heart dynamics could support more effective sleep enhancement outcomes and open a pathway toward accessible, non-invasive interventions to improve sleep quality and cognitive health.

## Graphical Abstract

**Graphical Abstract:**
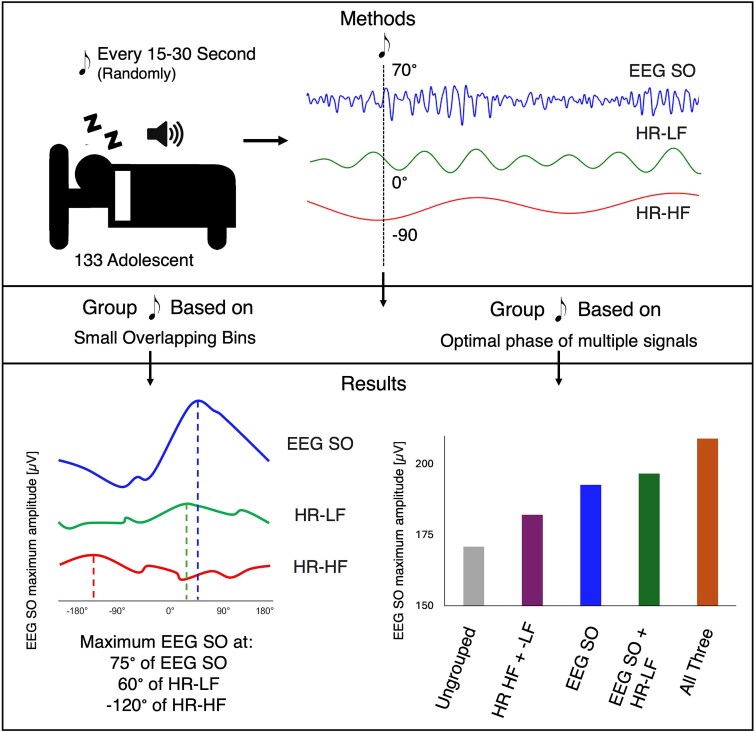

## Introduction

Non-rapid eye movement (NREM) sleep contributes to neural stability, supports psychological well-being, and maintains physiological homeostasis [1]. The deeper stages of NREM sleep are characterized by prominent neural synchronization, with slow oscillations (~0.8 Hz) representing the dominant synchronized EEG activity in NREM sleep [2]. Slow-wave activity (SWA; 0.5–4 Hz), which encompasses SOs, is closely linked to memory consolidation [3], synaptic homeostasis [4], and immune function [5], and also serves as a physiological marker of sleep’s restorative quality [6].

SWA naturally declines with age [7] and can also be reduced in individuals with physical or psychological health conditions [8]. Consequently, the enhancement of SOs and SWA has become a central focus of research aimed at improving sleep quality and associated cognitive and physiological outcomes.

A range of interventions has been explored, including invasive approaches such as pharmacological agents [9], non-invasive brain stimulation techniques such as transcranial direct current stimulation (tDCS) [10] and transcranial alternating current stimulation (tACS) [11], and behavioral strategies [12]. Other approaches include sensory stimulation modalities such as auditory or olfactory cues [13] delivered during sleep, with closed-loop auditory stimulation (CLAS)—in which brief tones are delivered in synchrony with specific neural activity phases—emerging as particularly promising due to its temporal precision and ease of implementation [14, 15].

Multiple studies have shown that auditory tone stimulation, when delivered during the SO upstate, maximizes SO amplitude and increases the likelihood of phase-locked spindle generation [16]. These stimulation-evoked dynamics have been linked to improved hippocampal–cortical communication and better performance on memory tasks, especially in declarative memory domains [17]. Experimental findings suggest that the peak of the SO upstate represents the most effective time for stimulation due to heightened cortical excitability at that phase [18]. Most CLAS systems, however, rely solely on EEG-based SO phase to determine stimulation timing and largely ignore the influence of peripheral physiological dynamics—despite well-established evidence of central–autonomic coupling during sleep [19].

Auditory stimulation during sleep engages both cortical and autonomic systems. Tones that elicit SOs are accompanied by transient increases in HR, blood pressure, and sympathetic activity, followed by compensatory rebounds toward or below baseline levels [19, 20]. Conversely, the likelihood of SO occurrence depends on autonomic state: they are more frequent during parasympathetic dominance, such as when blood pressure decreases [21] and when pre-tone cardiac activity is low [20]. These findings highlight a bidirectional coupling between central and autonomic events. HR low-frequency (LF; 0.04–0.15 Hz) and high-frequency (HF; 0.15–0.4 Hz) oscillations provide accessible indices of autonomic state, with HF power reflecting parasympathetic activity and LF power capturing a mix of sympathetic, parasympathetic, and baroreflex influences [22].

Based on prior work [23] showing that both cortical excitability and autonomic state shape the brain’s responsiveness to auditory input—and that specific autonomic phases, particularly the HR-LF upstate and HR-HF downstate, modulate this responsiveness—we formulated two directional hypotheses. First, extending earlier findings from binary upstate/downstate comparisons, we expected that tones occurring near the peaks of the HR-LF and HR-HF oscillations (ie, the HR-LF up-peak and HR-HF down-peak) would be associated with stronger enhancement of SO amplitude and SWA than tones occurring at other phases, as these peaks correspond to moments when autonomic oscillatory activity is most strongly expressed. Second, we hypothesized that combining EEG SO timing with HR-derived timing cues could show the largest enhancement effects, reflecting the additive contributions of cortical excitability and autonomic phase alignment in shaping tone-evoked slow-wave dynamics. To test these hypotheses, we investigated the optimal timing for auditory stimulation by examining which phases were associated with stronger responses based on continuous phase representations of EEG SOs, HR-LF, and HR-HF components. We also compared cortical responses evoked by tones occurred at the upstate and downstate phases of each oscillation, both individually and in combination. Specifically, we aimed to determine whether auditory stimulation occurring at specific HR component phases enhances SO amplitude and SWA more effectively than random delivery, and whether combining HR component phases with SO phase provides greater enhancement than EEG-based timing alone.

## Materials and Methods

### Participants and dataset

The research examined data from 133 adolescents (12–21 years old; mean ± SD: 15.6 ± 2.4 years; 60 females) from the National Consortium on Alcohol and NeuroDevelopment in Adolescence (NCANDA) [24] sleep substudy conducted at SRI International and the University of Pittsburgh. Previous studies [23, 25] provide complete details on participant demographics, enrollment procedures, Institutional Review Board (IRB) approval, site-specific sample sizes, and technical specifications of the polysomnography (PSG) setup. In summary, participants were healthy, drug-free, and without psychiatric, medical, or sleep disorders.

### Auditory stimulation and trial selection

Auditory tones (80 dB, 1000 Hz, 50 ms) were delivered binaurally during NREM sleep at random intervals of 15–30 s, as detailed in [23]. Analyses included only tones in N2 and N3 epochs free of artifacts (no EEG/ECG outliers within ±10 s) and arousals (none within ±10 s). Analyses were further restricted to trials that successfully evoked a SO; these 20-s segments were defined as the stimulated windows (STIM). Table 1 reports tones per individual per condition and the subset followed by a SO.

**Table 1:** Number of tones presented in each condition and number of tones followed by a SO (mean ± SD per individual)

**Condition** ^**a**^	**Tones Played**	**Tones → SO**
—	493.50 ± 163.58	324.62 ± 105.91
U—	339.81 ± 128.39	242.31 ± 97.57
–U–	331.94 ± 124.06	226.21 ± 84.98
—D	269.51 ± 98.90	180.66 ± 68.97
UU–	235.93 ± 104.70	172.21 ± 78.91
U–D	189.81 ± 82.36	137.77 ± 65.10
–UD	185.67 ± 78.27	129.36 ± 56.68
UUD	134.81 ± 67.98	100.36 ± 53.34

a Conditions are defined by phase-locking based on EEG SO, HR-LF, and HR-HF phases in order: U = upstate, D = downstate, – = no phase-locking

To serve as comparison trials, tone-free intervals of at least 20 s during N2 and N3 sleep were identified using the same artifact- and arousal-free criteria. Intervals whose midpoints were followed by a spontaneous SO were used as matched unstimulated windows (UNSTIM). These midpoints were treated as hypothetical tone-onset time points, enabling the assessment of spontaneous EEG activity under equivalent, non-stimulated conditions. Across participants, an average of 283.3 ± 127.2 STIM events were available, alongside 239.8 ± 177.5 UNSTIM windows.

### E‌EG preprocessing

EEG from the F3 electrode (referenced to the contralateral mastoid) was band-pass filtered between 0.1 and 30 Hz using a fourth-order Butterworth filter. The filter was applied in both directions to avoid phase shifts. Segments with amplitudes exceeding ±1500 μV or standard deviations greater than 10 from the mean were classified as artifacts and excluded [23].

**Figure 1:**
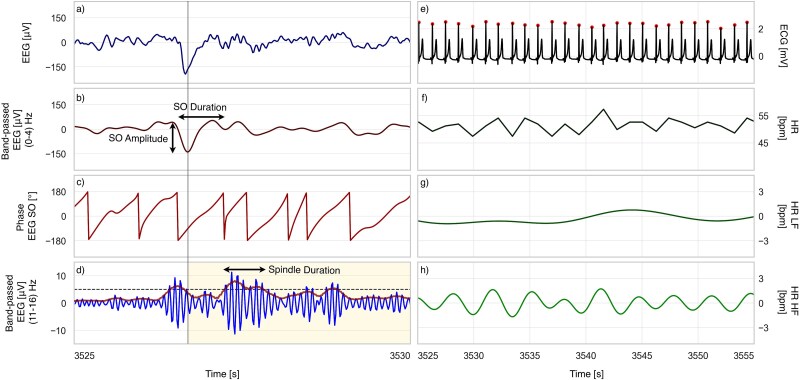
EEG and ECG signals with extracted oscillations. EEG-related panels: (a) raw EEG, (b) EEG filtered in the SO band with a sample SO showing its peak-to-peak amplitude and duration, (c) instantaneous phase of the EEG filtered in the SO band, and (d) EEG filtered in the spindle band with RMS envelope and spindle duration illustrated for a sample spindle. The shaded yellow area shows the search interval for SO-locked spindles. ECG-related panels: (e) raw ECG with detected R-peaks, (f) derived HR, (g) low-frequency (HR-LF) component, and (h) high-frequency (HR-HF) component. Each EEG panel shows a 5-s segment and each ECG panel a 30-second segment, both recorded during N3 sleep in a representative individual.

SOs were extracted by filtering the EEG between 0.5 and 4 Hz [26] using a fourth-order Butterworth filter. The SOs were defined as waves with a negative peak less than –80 μV, a peak-to-peak amplitude greater than 140 μV [27], and durations between 0.25 and 2.5 s. Peaks and troughs were identified between successive zero-crossings, and signal duration was measured between two downward zero-crossings (see Figure 1b).

To examine power alterations in specific frequency bands, EEG data were segmented into 8-second windows. Welch’s method was used to calculate power spectral density (PSD) with a 4-s Hanning window and 50 per cent overlap. The PSDs were then interpolated to a resolution of 0.1 Hz. To reduce inter-individual variability, PSDs for each participant were normalized to the mean total spectral power in the 0–20 Hz range across all stimulated windows. SWA power was defined as the mean power within 0.5–4 Hz [23].

Sleep spindle (SS) detection followed established criteria [28, 29]. EEG was band-pass filtered between 11 and 16 Hz, and the root mean square (RMS) envelope was computed with a 0.2-s sliding window. Candidate spindles were identified when the RMS envelope exceeded the 88.86th percentile of spindle activity during N2 and N3. Spindles were defined as events with lasting between 0.3 and 3 s, during which the RMS signal remained above threshold, containing at least five oscillations, a unimodal peak within the 11–16 Hz band, and decreasing power at higher frequencies, as determined using the Morlet wavelet. Stimuli-dependent spindles were those initiating after the trough of a detected SO following tone presentation (or its hypothetical timing in unstimulated windows). Finally, Spindle likelihood was defined as the proportion of stimuli-dependent spindles [18].

### ECG preprocessing

ECG signals were filtered using a fourth-order Butterworth band-pass filter (0.5–35 Hz). R-peaks were detected using custom algorithms [30], and R–R intervals were used to derive instantaneous HR, interpolated at 10 Hz. ECG segments with out-of-range signals or R–R intervals exceeding 10 standard deviations from the mean were excluded as artifacts or ectopic beats. HR signals were decomposed into LF (0.04–0.15 Hz) and HF (0.15–0.4 Hz) components [22] using separate fourth-order Butterworth filters.

### Phase-locking analysis

**Figure 2:**
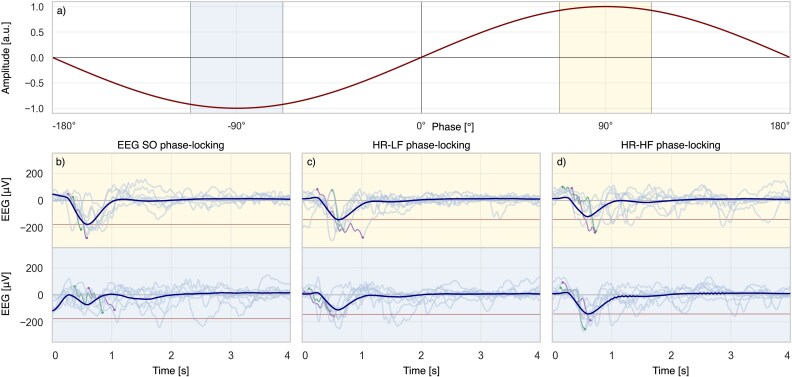
Sinusoidal signal and EEG responses to phase-locked stimuli. (a) Example sinusoidal signal with up-peak (yellow) and down-peak (blue) highlighted. (b–d) EEG averages time-locked to tones delivered at phases of (b) EEG SO, (c) HR-LF, and (d) HR-HF. Yellow shading indicates up-peak phase-locking and blue shading indicates down-peak phase-locking. The red horizontal line marks the lowest SO amplitude observed at the up- and down-peaks of each condition.

Instantaneous phases of EEG SOs and HR components (HR-LF and HR-HF) were computed using the Hilbert transform [21]. A 90° phase shift was applied to correct for quadrature offset, such that –90° corresponded to the trough, 90° to the peak, and 0° to the negative-to-positive zero crossing (see Figure 2a).

### Continuous phase analysis

To examine fine-grained phase effects, tones were grouped according to the instantaneous phase of EEG SO, HR-LF, and HR-HF at the time of tone onset. Phases were binned into 60° windows shifted by 15° across the full 360° cycle. Using overlapping bins helped reduce edge artifacts, and assumed that responses vary smoothly across neighboring phases. This design also ensured that each bin contained a sufficient number of tones, while the small shift preserved resolution.

For each tone trial in STIM and each hypothetical tone in UNSTIM, SO peak-to-peak amplitude, normalized SWA, and stimulus-dependent spindle occurrence were quantified. Monte Carlo (MC) baselines were estimated per phase bin (details in the Statistics section). SO amplitude and SWA were averaged within each phase bin, and spindle likelihood was defined as the proportion of SO-coupled spindles following tone presentation. These analyses yielded continuous indices describing how SO amplitude, SWA, and spindle likelihood varied across EEG SO, HR-LF, and HR-HF phases.

### Multidimensional phase analysis

EEG–HR phase-locking was analyzed using binary 180° bins. For each signal (EEG SO, HR-LF, HR-HF), the downstate was defined as –180° to 0° and the upstate as 0° to 180°. Phase-locking conditions were constructed either unimodally (based on one signal) or multidimensionally (combining EEG and/or HR components phases, eg, EEG upstate with HR-HF downstate).

For each condition, event-related potentials (ERPs) and PSDs were computed separately for STIM and UNSTIM by averaging tone-locked EEG segments. Difference signals were obtained by subtracting UNSTIM from STIM, producing difference waveforms and spectra. In the ERP domain, extrema were identified, and peak-to-peak amplitudes were quantified. In the spectral domain, PSDs were estimated from tone-locked segments, averaged within conditions, and compared to derive difference spectra. Mean SWA power (0.5–4 Hz) was extracted from all spectra.

### Statistics

#### Statistical analysis of continuous phase data

Responses were mean-centered within individuals to isolate phase-dependent effects. For SO amplitude and SWA, individual STIM and UNSTIM values were extracted for each phase bin. Because tone delivery was not uniformly distributed across phases, UNSTIM values were modeled by Gaussian approximations of empirical mean and variance. From this distribution, 200 Monte Carlo samples were generated to form phase-specific surrogate baselines. This approach allowed us to normalize each subject–bin by a common UNSTIM-derived reference without discarding data and ensured that STIM and UNSTIM differences reflected phase structure rather than unequal sampling. These provided STIM–MC and UNSTIM-MC contrasts at the individual level [18]. At the group level, one-sample *t*-tests compared STIM-MC and UNSTIM-MC across phase bins, with false discovery rate (FDR) correction applied using the Benjamini–Hochberg procedure (α = 0.05).

Spindle likelihood analysis used a Monte Carlo resampling approach to define a baseline. For each phase bin, UNSTIM events were resampled 200 times, drawing two-thirds of events per iteration. The geometric mean of these resamples likelihood provided a null reference value for that bin. For each participant, spindle likelihood was then calculated as the percentage of SO-coupled spindle occurrences in STIM and UNSTIM conditions, expressed relative to this baseline. Differences between STIM and UNSTIM were tested per bin using Welch’s unequal-variance *t*-tests, and *p*-values were corrected for multiple comparisons using the FDR method.

In addition, we computed a Pairwise Response Index (PRI) to quantify phase preference at the individual level. For each subject, we considered the distribution of STIM–MC contrasts in each phase bin, obtained from the Monte Carlo procedure described above. For every pair of bins, we compared the corresponding STIM–MC distributions using Welch’s unequal-variance *t*-test and noted whether the t-statistic was positive or negative. The PRI for a given bin was defined as the average sign of these pairwise t-statistics across all comparisons with other bins, yielding values between −1 (consistently weaker than other bins) and + 1 (consistently stronger than other bins). Group-level PRI values were then tested against zero using one-sample *t*-tests per bin, and *p*-values were corrected for multiple comparisons using FDR (α = 0.05).

#### Statistical analysis of multidimensional phase data

For binary phase comparisons (upstate versus downstate; EEG–HR combinations), individual-level averages from STIM, UNSTIM, and their differences (STIM-UNSTIM) were compared across phase-locking strategies. Normality was assessed with the Shapiro–Wilk test (α = 0.05). Paired-samples *t*-tests were used when normality held; otherwise, Wilcoxon signed-rank tests were applied.

#### Software

Preprocessing was performed in MATLAB R2023a (MathWorks, Natick, MA). Statistical analysis and visualization were conducted in Python 3.9.

## Results

### Tone presentation was optimal at SO up-peak, HR-LF up-peak, and HR-HF down-peak.

**Figure 3:**
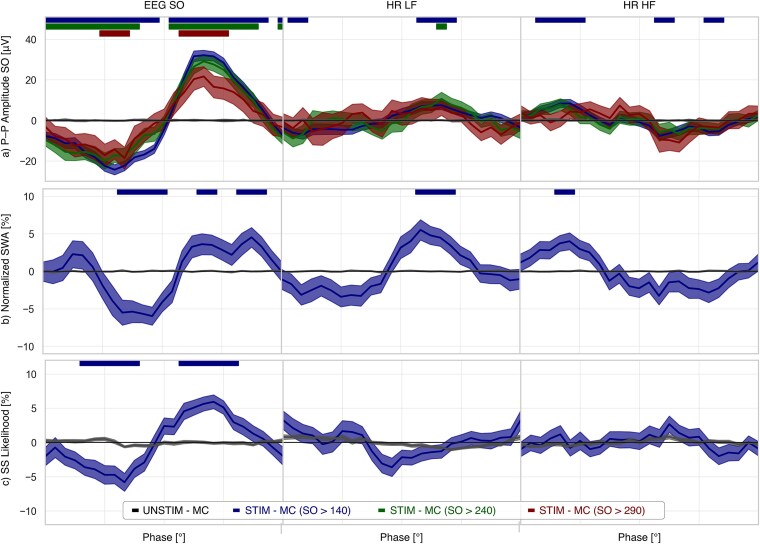
Continuous SO amplitude, SWA, and spindle likelihood as a function of SO and HR component phases. (a) SO peak-to-peak amplitude across EEG SO, HR-LF, and HR-HF phases. (b) Continuous SWA and (c) continuous sleep-spindle likelihood across phases. Phases are mapped from –180° to 180°, and shaded ribbons indicate phase intervals where tone-evoked responses differed significantly from unstimulated intervals.

Continuous phase analysis (Figure 3) showed phase-dependent differences across EEG SO, HR-LF, and HR-HF. Peak-to-peak amplitudes were largest when tones were delivered at the SO up-peak, HR-LF up-peak, and HR-HF down-peak, and smallest at the SO down-peak, HR-LF down-peak, and HR-HF up-peak. The windows of significant differences in the PRI comparison were similar to those found in the STIM-MC vs. SHAM-MC analysis.

As in Figure 3a, the maximum SO amplitude occurred at 75° (*p* < .001), 60° (*p* < .05), and −120° (*p* < .001) for SO, HR-LF, and HR-HF, respectively. Minimal responses were at −75° (*p* < .05), −150° (not significant), and 30° (*p* < .01). Differences between extreme bins reached 55.6 μV for SO (*p* < .001), 14.5 μV for HR-LF (*p* < .001), and 15.9 μV for HR-HF (*p* < .001). Windows of significant effects spanned 225° and 195° for SO (optimal and non-optimal phases), 105° and 75° for HR-LF, and 120° and 75° for HR-HF. Larger SOs were less responsive to stimulation and were influenced only by SO phase. Medium SOs were influenced by SO and HR-LF timing. Small SOs were most sensitive, showing phase-dependent changes across all oscillatory components.

SWA showed similar patterns (Figure 3b). Largest increases were at 135° (*p* < .01), 30° (*p* < .01), and −120° (*p* < .01), with minima at 0° (*p* < .01), −90° (not significant), and 30° (not significant) for SO, HR-LF, and HR-HF. Significant windows covered 60° (optimal) and 120° (non-optimal) for SO, 105° for HR-LF, and 75° for HR-HF. SWA modulation magnitudes, computed as the difference between extremes relative to mean SWA across individuals, were 10.6 per cent for SO, 8.9 per cent for HR-LF, and 6.9 per cent for HR-HF (Figure 1b).

Spindle likelihood (Figure 3c) was phase-dependent for SO but not for HR components. Relative to the mean across bins, spindle likelihood was enhanced near 75° (*p* < .001) and suppressed near −60° (*p* < .001). The associated windows measured 135° and 135°, and the difference between extremes was 11.2 per cent (*p* < .001).

### Peak HR response occurred at the SO up-peak.

**Figure 4:**
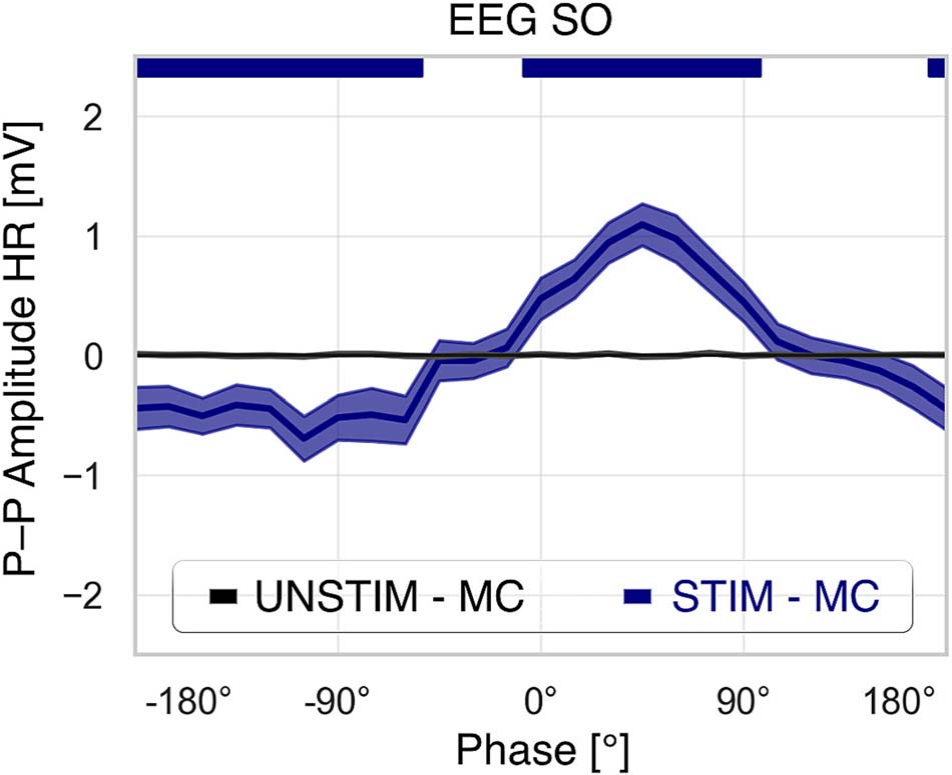
Continuous HR peak-to-peak amplitude as a function of SO phases. Phases are mapped from –180° to 180°, and shaded ribbons indicate phase intervals where tone-evoked responses were significantly different from unstimulated intervals.

Cortical–cardiac continuous phase analysis (Figure 4) showed HR responses were phase-dependent on EEG SO activity. HR increased most during the ascending positive SO phase at 45° (*p* < .001) and decreased during the descending negative phase at −105° (*p* < .001). Windows covered 150° and 180°, respectively. The difference between extreme bins was 1.8 bpm (*p* < .001).

### SO and SWA were enhanced by HR component phase-locking alone

**Figure 5:**
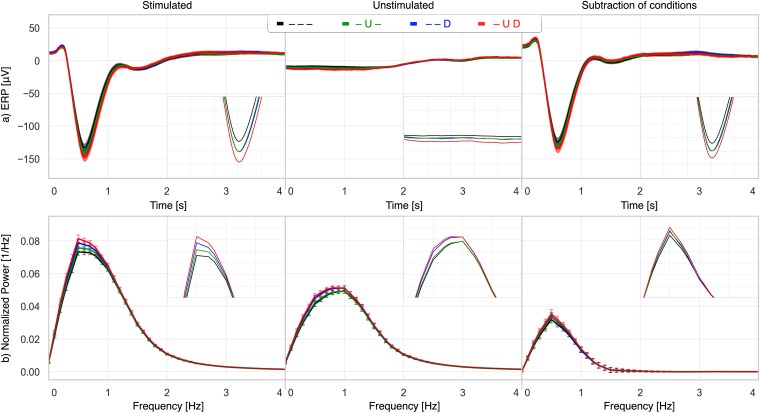
Average EEG and PSD based on HR component phase-locking and multidimensional HR component combinations. From left to right: Stimulated, unstimulated, and their difference. (a) EEG time-locked to tones. (b) PSD up to 4 Hz. Across all panels, placeholders remain consistent: “–” denotes no phase-locking to that signal, while “U” and “D” indicate upstate and downstate, respectively. For example, the legend “—D” indicates no phase-locking to EEG SO or HR-LF, with tones phase locked to the HR-HF downstate.

In multidimensional phase analysis, cardiac contributions were assessed by comparing EEG responses between open-loop stimulation (across all phases) and stimulation phase-locked to HR-LF upstate and/or HR-HF downstate (Figure 5; Table 2). Data from 104 participants with sufficient tone counts were analyzed.

**Table 2:** SO amplitude and SWA for stimulated and unstimulated conditions across different phase-locking strategies

**Condition**	**Phase-locking based on**			**Metrics (stimulated)**		**Metrics (unstimulated)**	
	**EEG SO**	**HR-LF**	**HR-HF**	**SO Amplitude (μV)**	**SWA** ^*^ **(%)**	**SO Amplitude (μV)**	**SWA (%)**
Open-loop	✗	✗	✗	170.80	123.22	12.06	100
HR phase-locking 1	✗	✓^†^	✗	180.47	127.82	24.84	103.86
HR phase-locking 2	✗	✗	✓	177.87	124.61	24.78	99.59
HR phase-locking 3	✗	✓	✓	187.58	129.24	35.46	103.07
SO phase-locking 1	✓	✗	✗	208.63	124.12	80.256	99.42
SO phase-locking 2	✓	✓	✗	218.59	128.60	87.772	103.50
SO phase-locking 3	✓	✗	✓	215.72	124.97	88.382	98.17
SO phase-locking 4	✓	✓	✓	226.20	129.15	98.552	101.92

^*^Expressed as percentage of the average SWA in unstimulated windows without phase-locking

^†^Tick mark indicates conditions where stimulation was phase-locked to the optimal state of the corresponding oscillation

Relative to random stimulation, tones presented during HR-LF upstate, HR-HF downstate, or combined HR-LF + HR-HF increased SO amplitude by 11.2 μV (*p* < .001), 10.1 μV (*p* < .001), and 21.8 μV (*p* < .001). SWA increased by 4 per cent (*p* < .01), 7 per cent (*p* < .001), and 12 per cent (*p* < .001). Pairwise tests showed significant differences between HR-LF and the combined condition (SO *p* < .001; SWA *p* < .01) and between HR-HF and the combined condition (SO *p* < .01; SWA *p* < .05). No significant differences were observed between HR-LF and HR-HF alone.

### E‌EG–HR phase-locking enhances SO and SWA beyond EEG alone.

**Figure 6:**
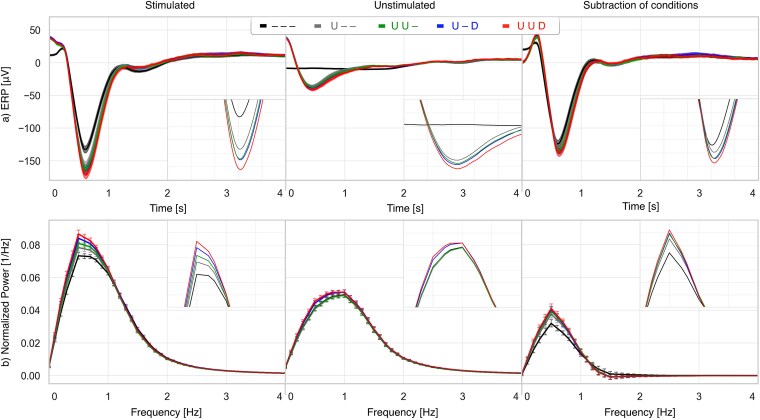
Average EEG and PSD based on EEG SO phase-locking and multidimensional EEG–HR component combinations. From left to right: Stimulated, unstimulated, and their difference. (a) EEG time-locked to tones. (b) PSD up to 4 Hz. Across all panels, placeholders remain consistent: “–” denotes no phase-locking to that signal, while “U” and “D” indicate upstate and downstate, respectively. For example, the legend “U–D” indicates phase-locking to the SO upstate and the HR-HF downstate, with no phase-locking to HR-LF.

The multidimensional phase analysis was further extended to EEG–HR combinations by comparing stimulation effects when tones were phase-locked to the SO upstate alone versus when combined with HR-LF upstate and/or HR-HF downstate (Figure 6). Versus random stimulation, phase-locking to SO upstate alone increased SO amplitude by 17.8 μV (*p* < .001) and SWA by 19 per cent (*p* < .001). Adding HR-LF increased responses to 27.3 μV (*p* < .001) and 23 per cent (*p* < .001). Adding HR-HF yielded 25.7 μV (*p* < .001) and 28 per cent (*p* < .001). The strongest effects occurred with simultaneous SO upstate, HR-LF upstate, and HR-HF downstate: 38.1 μV increase in SO amplitude (*p* < .001) and 32 per cent increase in SWA (*p* < .001).

Comparisons across combinations showed no significant differences between SO+HR-LF or SO+HR-HF relative to SO alone (SO not significant; SWA not significant). However, the full triple-locking condition produced significantly greater SO and SWA enhancement than SO combined with either HR component alone. Specifically, SO+HR-LF versus SO+HR-LF + HR-HF differed significantly (*p* < .001 for SO; *p* < .001 for SWA), and SO+HR-HF versus SO+HR-LF + HR-HF also differed (*p* < .001 for SO; SWA not significant).

## Discussion

This study demonstrates that peripheral oscillatory phases, particularly HR-LF and HR-HF dynamics, carry timing information that may be useful for optimizing CLAS. By evaluating the full phase continuum and comparing unimodal and combined EEG–HR strategies, we found that tones that occurred near specific HR phases were associated with larger enhancements in SO amplitude and SWA, and that the strongest effects emerged when SO up-states coincided with these autonomic optima. These findings extend prior CLAS work, which has predominantly relied on EEG-only cues, by highlighting a potential role for brain–heart coupling in shaping the timing-related effectiveness of stimulation.

Consistent with prior research, our results show that the timing of auditory stimulation critically determines the extent of SO and SWA enhancement [31]. While SO-based phase-locking is well established, our study introduces novelty by identifying precise HR-HF and HR-LF phases that also modulate SO amplitude and SWA. These findings indicate that auditory stimulation systems can leverage HR component phases either independently or in combination with EEG SO, providing a multidimensional approach to phase-locked stimulation.

We observed that SO amplitude increased when tones occurred during the rising phase of SOs, particularly near the up-peak, consistent with prior findings [18]. Spindle likelihood and SWA also increased at nearly the same phases, indicating that this SO timing represents a general optimal target. Beyond EEG SOs, tones applied to the up-peak of HR-LF and the down-peak of HR-HF also enhanced responses.

Similar to earlier studies [18], the effective windows were narrower (by 75° at peak) for large SOs (>290 μV) compared to smaller SOs (>140 μV) when phase-locking was based on EEG SOs. In our study, this was also accompanied by a reduced amplitude of post-stimulus increases for large SOs. Moreover, we observed that large SOs could not be enhanced using peripheral rhythms. This pattern suggests that once SOs reach higher amplitudes, their dynamics approach a physiological ceiling, limiting the extent to which external stimulation—whether timed using cortical or autonomic rhythms—can further modulate them. Consequently, effective enhancement of large SOs may require exceptionally precise timing, and autonomic rhythms alone may not provide sufficient modulatory influence.

Spindle likelihood showed clear phase dependence with EEG SOs but not with HR components. Because memory consolidation depends in part on SO–spindle coupling [32], the lack of spindle enhancement suggests that HR-based timing may be less predictive of memory outcomes. However, SO amplitude alone has been shown to predict next-day memory improvements [33]. Thus, experiments implementing HR-based timing of SO stimulation with pre- and post-sleep memory testing are needed to evaluate memory benefits explicitly and to determine how they compare with EEG-based timing.

Importantly, the results demonstrate that SO amplitude and SWA enhancement can be achieved through HR component phase-locking, either independently or in combination with EEG SO. Effects scaled systematically: modest increases with HR-LF or HR-HF alone (≤11.2 μV in SO amplitude; ≤7% in SWA), moderate with both HR components combined (21.8 μV; 12%), stronger when pairing one HR component with EEG SO (≤25.7 μV; ≤28%), and maximal when integrating both HR components with EEG SO (≤38.1 μV; 32%). These findings highlight the potential of HR-guided closed-loop stimulation strategies for wearable systems where EEG recordings are challenging, but ECG is readily available. They also demonstrate the value of hybrid strategies, which yield maximal enhancement when both EEG and ECG can be acquired.

Theoretically, our findings show that cardiac oscillations may influence sleep dynamics alongside cortical activity [19]. This supports the view that the autonomic system actively participates in processing external inputs [34], which aligns with network-based models in which brain and body oscillations interact as components of a dynamic system [35]. From this perspective, auditory stimulation not only modifies local brain rhythms but also transiently reorganizes large-scale cortico–autonomic coupling [14].

Prior work has shown interactions between cardiac activity and sigma oscillations—for example, increases in sigma power following autonomic events and transient autonomic changes accompanying spindle occurrences [36]. However, these findings describe event-related co-modulation rather than a stable, cycle-by-cycle alignment between spindles and the ongoing phase of HR-LF or HR-HF rhythms, and thus do not imply that timing stimulation to HR-component phases should enhance SO-coupled spindle expression. Consistent with this distinction, we did not observe modulation of SO-coupled spindle likelihood across HR-LF or HR-HF phases. Nonetheless, other ECG-derived features may contain more precise autonomic signatures associated with spindle generation and could provide more effective timing cues for closed-loop stimulation. Therefore, future work should explore alternative ECG-based indicators—beyond the phase of HR-LF and HR-HF—to identify the most effective peripheral timing cues for modulating SO-coupled spindle activity.

A limitation of the present approach is the absence of a separate control night. Although tone-free segments within each night (UNSTIM windows) provided closely matched comparison periods, this approach cannot fully exclude the possibility of longer-lasting effects of acoustic stimulation. In addition, UNSTIM windows were not explicitly balanced across sleep cycles. Future studies incorporating dedicated sham-control nights and ensuring cycle-matched sampling of STIM and UNSTIM events will be important for isolating stimulation effects and refining phase-specific comparisons.

An important limitation of this study relates to the distribution of tones across conditions. Random delivery of tones every 15–30 s created uneven distributions across bins. This imbalance was worsened by the lower SO induction rates in non-optimal phases, which reduces available events for comparison. Future studies should adopt shorter or structured intervals, ideally combined with control nights, so that no tone-free interval is needed, and more balanced tone distributions can be achieved.

Stimulus characteristics also play a critical role. In this study, we used a dataset that employed brief auditory tones (50 ms, 1000 Hz) at 80 dB, following a standard ERP-style parameter described by Colrain et al. [37]. In contrast, many CLAS studies employ brief (~50 ms), low-intensity (40–60 dB) pink noise bursts, which may more effectively enhance SOs while minimizing arousal. Comparative studies are needed to test how different stimulus parameters influence both cortical and autonomic outcomes.

Additionally, although SO amplitude, SWA, and spindle likelihood are strongly linked to memory consolidation, direct cognitive testing is necessary to confirm functional benefits. This is particularly relevant because HR component phase-locking increased SO amplitude and SWA but did not enhance spindle likelihood. Future studies should therefore incorporate declarative and procedural memory tasks as well as measures of attention and executive function to determine whether the observed physiological changes translate into cognitive gains.

Multidimensional phase-locking raises another challenge. While combining HR and EEG SO phases produced stronger enhancements, it also reduced the number of tones per condition. This raises the question of whether stimulation is more effective when delivering more frequent but weaker effects, or when delivering fewer tones with stronger effects. A practical solution is to develop predictive algorithms that adaptively select the best available phase-locking option in real time, ensuring tone counts remain high while exploiting multidimensional phase information. In addition, including nights without tone application would help identify how often such optimal opportunities occur naturally and how they differ from stimulated nights.

For future work, it will be essential to deliver stimulation at specific EEG and HR phases rather than relying on post-hoc analyses. Prospective phase-targeting would not only allow causal testing of the effects observed here but also provide an opportunity to evaluate the practical challenges and yield of targeting these oscillations in real time. Although methods for predicting ECG R-peaks exist, algorithms capable of accurately predicting HR-derived phases remain limited, and the feasibility of real-time LF- and HF-phase estimation has yet to be demonstrated. Because HF-HR oscillations typically have cycle durations of approximately 2–7 s and LF-HR oscillations evolve even more slowly (around 7–25 s per cycle), these autonomic rhythms may be easier to target once an instantaneous phase is identified. However, systematic testing will be required to determine how reliably these phases can be predicted and applied in closed-loop stimulation.

Beyond these limitations, these results suggest that integrating peripheral signals into closed-loop algorithms offers a promising direction. Wearable devices that combine EEG with HR components could use the optimal phases to enhance SO and SWA through cumulative effects. Such multimodal strategies may reduce inter-individual variability and facilitate the translation of stimulation protocols into practical sleep-enhancement technologies.

## Conclusions

Auditory stimulation was associated with increases in SO amplitude and SWA, and these effects varied systematically with oscillatory phase. The largest responses occurred when tones happened to fall near the SO up-peak, the HR-LF up-peak, and the HR-HF down-peak. Multidimensional phase analyses further showed that cardiac timing carried informative structure, with the strongest enhancements observed when EEG and HR phase optima coincided. These results highlight coordinated CNS–ANS dynamics in sleep neurostimulation and suggest that future closed-loop systems may benefit from multidimensional phase-targeting to optimize sleep physiology and support wearable applications for enhancing memory and resilience.

## Data Availability

Data are available from the corresponding author upon reasonable, non-commercial request.
